# Myocardial *DYRK1B* Expression Is Increased in Patients with Impaired Cardiac Contractility and Sleep-Disordered Breathing

**DOI:** 10.3390/antiox14020163

**Published:** 2025-01-29

**Authors:** Fatma Bayram, Philipp Hegner, Anna-Maria Lauerer, Sönke Schildt, Dominik Wermers, Maria Johanna Baier, Julian Mustroph, Maria Tafelmeier, Zdenek Provaznik, Christof Schmid, Lars Siegfried Maier, Stefan Wagner, Michael Arzt, Simon Lebek

**Affiliations:** 1 Department of Internal Medicine II, University Hospital Regensburg, 93053 Regensburg, Germany; fatma.bayram@ukr.de (F.B.); philipp.hegner@ukr.de (P.H.); anna-maria.lauerer@klinik.uni-regensburg.de (A.-M.L.); soenke.schildt@ukr.de (S.S.); dominik.wermers@ukr.de (D.W.); maria.baier@ukr.de (M.J.B.); julian.mustroph@ukr.de (J.M.); maria.tafelmeier@ukr.de (M.T.); lars.maier@ukr.de (L.S.M.); stefan.wagner@ukr.de (S.W.); michael.arzt@ukr.de (M.A.); 2 Department of Cardiothoracic Surgery, University Hospital Regensburg, 93053 Regensburg, Germany; zdenek.provaznik@ukr.de (Z.P.); christof.schmid@ukr.de (C.S.)

**Keywords:** DYRK1B, heart failure, sleep-disordered breathing, cardiovascular disease, hypoxia

## Abstract

Heart failure and cardiovascular disease represent a significant burden on healthcare systems worldwide. Recent evidence associates an increased expression of the dual-specificity tyrosine phosphorylation-regulated kinase 1B (DYRK1B) with an impaired cardiac function in mice. However, there remains a paucity of data on myocardial *DYRK1B* expression in patients with cardiovascular disease in the context of other comorbidities. In our study, we examined *DYRK1B* mRNA expression in human right atrial appendage biopsies from 159 patients undergoing elective coronary artery bypass surgery. Each patient was tested for sleep-disordered breathing the night prior to surgery. In this large representative study cohort with cardiovascular high-risk patients, we found that an impaired cardiac function as well as sleep-disordered breathing (SDB), including various oxidative stress parameters, were associated with an increased myocardial *DYRK1B* expression. A multivariate regression analysis revealed left ventricular ejection fraction and the presence of SDB as significant predictors of the myocardial *DYRK1B* expression independent of other clinical covariates. Based on these findings, DYRK1B represents a promising molecular target in patients with heart failure and reduced ejection fraction as well in patients with sleep-disordered breathing.

## 1. Introduction

Cardiovascular disease remains the leading cause of death with a decades-long increase in most of the countries worldwide [1]. Heart failure (HF) represents a major global healthcare issue and the hospitalization as well as mortality rates remain high [2,3]. Despite ongoing progress in therapeutic developments (e.g., angiotensin receptor/neprilysin inhibitors), there was only limited success in improving the survival of heart failure patients as the current 5-year survival rate is 45.5% after initial diagnosis [3]. While present therapeutic strategies are often not fully effective, they are also frequently associated with relevant adverse side effects [4]. The latter typically decreases the patients’ compliance to take their prescribed medications, which decreases the overall treatment success [5]. Thus, further research and identifying potential molecular targets are urgently needed to develop new therapeutic approaches.

The dual-specificity tyrosine phosphorylation-regulated kinases (DYRKs) are part of the CMGC kinases, which are named after the initials of its core members (i.e., Cyclin-dependent kinases (CDKs), Mitogen-activated kinases (MAPs), Glycogen synthase kinases (GSKs), and CDK-like kinases (CLKs)) [6]. DYRK kinases play an important role in cell proliferation, differentiation, and signal transduction by phosphorylating specific serine, threonine, and tyrosine residues [7]. They share a highly conserved kinase domain, whereas the C-terminal structure is different, which explains the various functions across the DYRKs [8]. Among others, they differ in substrate specificity and their subcellular localization [9]. The five described mammalian DYRK isoforms are further classified into class I, represented by DYRK1A and DYRK1B, and class II, represented by DYRK2, 3, and 4 [10]. Class I DYRKs are mainly found in the nucleus, while class II DYRKs are predominantly localized in the cytoplasm [11].

DYRK1A and DYRK1B show a 85% homology in their kinase domain, which is why they are referred to as paralogous kinases [12]. Both kinases play an important role in the cell cycle [13]. DYRK1A is the best-characterized kinase within the family [14]. Increased *DYRK1A* expression was described in Down syndrome, while elevated *DYRK1B* levels were reported in solid tumors, such as gastrointestinal tumors or lung cancer [9], and acts as a negative cell cycle regulator in many tissues (e.g., by enhancing the resistance of cancer cells to chemotherapy) [15]. DYRK1B kinase represents a significant pharmacological target in cancer therapy due to its frequent overexpression in various tumors, whereby elevated *DYRK1B* levels are associated with poor clinical outcomes and reduced survival rates in patients [15].

Only little is known about the impact of DYRK1B on cardiac function and contractility. A recent article by Zhuang et al. demonstrated that *DYRK1B* levels were elevated in hypertrophic mouse hearts, and its overexpression led to cardiac impairment, along with a reduced left ventricular ejection fraction (LVEF) [16]. Notably, the authors found a remarkable upregulation of myocardial *DYRK1B* expression in mice with afterload-induced heart failure in a model of transverse aortic constriction (TAC), whereby the *DYRK1B* knockout mice remained protected from HF development [16].

The kinase is linked not only to cardiac dysfunction but also to metabolic syndrome [16,17]. Gain-of-function *DYRK1B* mutations promoted increased glucose production and enhanced adipogenesis [17]. Moreover, obesity is strongly linked to the presence of sleep-disordered breathing (SDB) [18], suggesting an interconnected relationship between *DYRK1B* activity, cardiac dysfunction, obesity, and sleep-disordered breathing.

Our goal was to investigate myocardial *DYRK1B* expression in 159 clinically well-characterized patients with high cardiovascular risk. We found that patients with an impaired cardiac contractility showed a significantly increased myocardial *DYRK1B* expression. Furthermore, the presence of SDB was independently linked to elevated *DYRK1B* expression, regardless of body mass index or LVEF.

## 2. Methods and Materials

### 2.1. Study Design

In our cross-sectional study, we analyzed patients who were enrolled in the study “Rationale and design of the CONSIDER AF study—Impact of sleep-disordered breathing on atrial fibrillation and perioperative complications in patients undergoing coronary artery bypass grafting surgery—a prospective observational study” [19]. This study was approved by the local ethics committee (University of Regensburg, Bavaria, Germany; 15-238-101) and conducted in accordance with the Declaration of Helsinki (most recent revision in 2013). Each patient provided written informed consent prior to inclusion. Only upon justified request and after written informed consent has been obtained from each patient included in the study, patient data can be made available.

**Figure 1:**
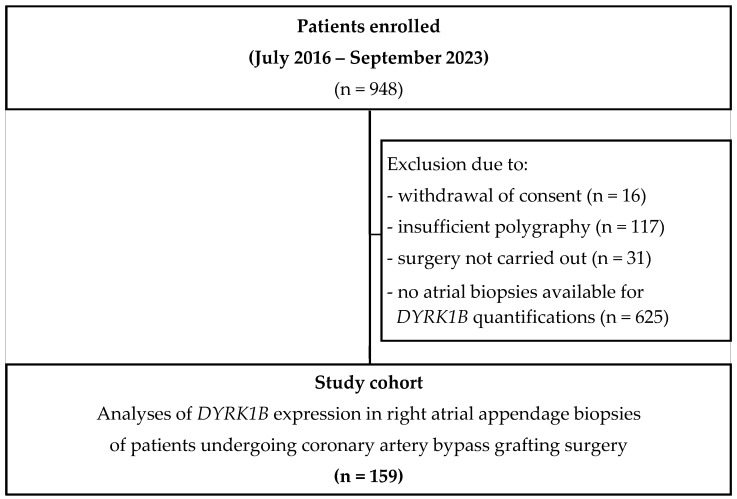
Flowchart of patients’ inclusion.

Patients undergoing elective coronary artery bypass grafting (CABG) surgery at the University Hospital Regensburg between July 2016 and September 2023 were included in the study if they were between 18 and 85 years old and had provided their written consent for participation. Patients with preexisting treated sleep apnea, mechanical ventilation or nocturnal positive airway pressure support therapy, home oxygen therapy, severe obstructive pulmonary disease, and/or preoperative use of an intra-aortic balloon pump or inotropes were excluded. Out of the 948 patients screened as eligible, the final number of patients included in this sub-study cohort was 159. Among the excluded patients, surgery was not performed in 31 cases, 16 patients withdrew their consent, 117 had insufficient polygraphy, and there was no myocardial biopsy available for *DYRK1B* quantification from 625 patients (Figure 1). Human right atrial appendage biopsies were obtained during the surgery, transported on ice in the cardioplegic solution Custodiol^®^ (2 mmol/L butanedione monoxime), and used for experimental analyses by blinded investigators. In addition, we obtained ventricular samples from 6 patients.

### 2.2. Polygraphic Assessment

As previously described in Tafelmeier et al., CONSIDER-AF’s design was structured to ensure that the night prior to surgery, each patient was tested for SDB by standardized polygraphy (Alice NightOne device; Philips Respironics, Murrysville, PA, USA) [19]. Respiratory effort was measured via respiratory inductance plethysmography, blood oxygen levels were monitored with a pulse oximetry, and airflow was measured with a nasal pressure cannula. Hypopneas were defined as a reduction of ≥30–90% in airflow versus baseline, lasting for at least 10 s, while an apnea was defined as a ≥90% reduction in airflow for ≥10 s. Desaturations were defined as a reduction in oxygen saturation of ≥4%. The average frequency of hypopneas and apneas per hour was used to calculate the apnea–hypopnea index (AHI). SDB was diagnosed by an AHI ≥ 15/h. The oxygen desaturation index (ODI) was calculated based on the frequency of desaturations per hour of sleep.

### 2.3. Quantification of DYRK1B mRNA Expression

RNA was isolated from right atrial appendage biopsies using the RNeasy Mini Kit (Qiagen, Venlo, Netherlands, catalog number 74106). To transcribe 1 µg RNA into cDNA, random primers (Promega, Madison, WI, USA, catalog number C1181), RNasin^®^ ribonuclease inhibitor (Promega, catalog number N2115), reverse transcriptase (Promega, catalog number M170B), reverse transcriptase 5x reaction buffer (Promega, catalog number M531A), and PCR nucleotide mix (Promega, catalog number C1145) were incubated for 1 h at 37 °C.

Gene expression was quantified using a TaqMan™ Fast Advanced Master Mix (Applied Biosystems, Waltham, MA, USA, catalog number 4444557) and pre-designed TaqMan^®^ Gene Expression Assays (Applied Biosystems) for *DYRK1B* (assay ID: Hs01043777_m1), *SOD1* (assay ID: Hs00533490_m1), *SOD2* (assay ID: Hs00167309_m1), *CAT* (assay ID: Hs00156308_m1), and the housekeeper gene *ACTB* (assay ID Hs00357333_g1). Real-time qPCR with cDNA was performed on a ViiA 7 real-time PCR system (Applied Biosystems). Following an initial uracil-N-glycosylase incubation at 50 °C (2 min) and polymerase activation at 95 °C (2 min), 40 cycles of 95 °C (1 s) and 60 °C (20 s) were performed. The average threshold cycle (Ct) was determined by measuring each sample in triplicates. This value was used for the comparative threshold cycle (Ct) relative quantification analysis method. To determine the individual delta-Ct value (dCt), the mean Ct value of the housekeeper gene *ACTB* was subtracted from the mean Ct value of the target gene. The relative mRNA expression as percentage of *ACTB* was determined by using the formula 2^−dCt^ × 100.

### 2.4. Statistics and Data Analyses

All experiments were conducted and analyzed blinded to the clinical data. The clinical data are presented as mean ± standard deviation (SD) or as total number of patients (with relative proportion), whereas experimental data are presented as mean per patient ± standard error of the mean (SEM).

A Shapiro–Wilk normality test was used to test for normal distribution. For data analyses and comparisons between the two groups, a Student’s t test was used for normally distributed continuous variables and Wilcoxon–Mann–Whitney test for non-normally distributed continuous variables. A Chi-square test was applied to analyze categorical variables. A Fisher’s exact test was applied for categorial variables with low expected frequencies. An ANOVA with a Holm–Sidak’s post hoc multiple comparisons test was used when comparing more than two groups. Univariate linear regression analyses were conducted to test for correlations. To test for the risk of an increased *DYRK1B* expression (≥median; dependent variable), we performed univariate and multivariate logistic regression analyses with various clinical risk factors as independent variables. Two-sided *p*-values below 0.05 were considered statistically significant.

## 3. Results

### 3.1. Baseline Characteristics and Polygraphy Dates of the Study Cohort

As shown in Table 1, our study cohort was a typical cardiovascular high-risk population with a mean age of 66.1 ± 8.7 years and 136 (86%) male patients. The average BMI was 28.5 kg/m^2^ with a mean left ventricular ejection fraction of 54.1 ± 10.4%. A total of 108 (68%) patients had hyperlipidemia, and 133 (84%) study participants were hypertensive. In total, 115 (72%) participants had an NYHA functional class > I.

As patients with high cardiovascular risk frequently suffer from SDB, we tested each patient with polygraphy in the night prior to surgery [20]. With a mean total recoding time (TRT) of 481.9 ± 47.6 min, SDB was diagnosed in 80 (50%) patients. Additionally, for further analysis, data from various hypoxia parameters, such as mean or minimum oxygen saturation, were evaluated (Table 2).

Based on the median *DYRK1B* expression level (1.098% of *ACTB*), the total study cohort was split for further analyses into two sub-cohorts: the first group with *DYRK1B* levels below the median (n = 79) and the second group with *DYRK1B* levels greater than or equal to the median (n = 80). In addition, we provide further insights into the patients’ characteristics, based on the LVEF and existing SDB, in Supplemental Table S1.

Further analyses were conducted to evaluate postoperative outcomes. Although patients with a high *DYRK1B* expression tended to experience more frequently postoperative atrial fibrillation and major adverse cardiac cerebrovascular events and had longer postoperative hospital stays, these differences did not reach statistical significance (Supplemental Table S2).

### 3.2. Impaired Cardiac Contractility in Patients with Elevated DYRK1B Levels

In our analysis, patients with increased *DYRK1B* expression levels showed several significant features of heart failure with reduced ejection fraction (HFrEF), while there were no differences in age, gender distribution, BMI, diabetes mellitus, atrial fibrillation, renal function, coronary artery status, or patient treatment (Table 1).

**Figure 2:**
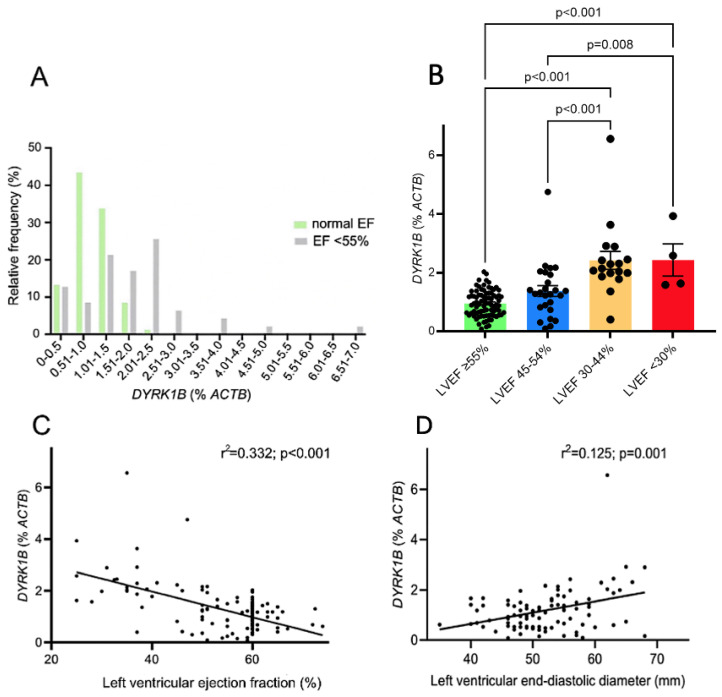
*DYRK1B* expression is increased in right atrial appendage biopsies of patients with a reduced left ventricular ejection fraction. (**A**) Frequency distribution of patients with a normal and a reduced left ventricular ejection fraction (LVEF), based on the *DYRK1B* expression. (**B**) Mean *DYRK1B* expression in different LVEF sub-categories. (**C**) Linear regression analysis between the LVEF and the corresponding *DYRK1B* expression. (**D**) Linear regression analysis between the left ventricular end-diastolic diameter and the corresponding *DYRK1B* expression.

Figure 2A illustrates the frequency distribution of patients with a normal (≥55%) or a reduced (<55%) LVEF, based on the *DYRK1B* expression. It is evident that a reduced LVEF was more prevalent when *DYRK1B* was increased. In fact, patients with elevated *DYRK1B* expression levels had a lower LVEF and an increased left ventricular end-diastolic diameter (LVEDD) compared to participants with low *DYRK1B* levels (Table 1). We then analyzed patient sub-categories based on the LVEF range (Figure 2B). Notably, the data indicate a marked increase in *DYRK1B* expression when the LVEF is below 45%. We found a >3-fold-increased myocardial *DYRK1B* expression in patients with a LVEF of 30–44% and with a LVEF below 30% compared to patients with a normal LVEF ≥ 55% (*p* < 0.001). This resulted in a significant negative correlation between the LVEF and *DYRK1B* expression (r^2^ = 0.332, *p* < 0.001, Figure 2C). Similarly, there was a significant positive correlation between the LVEDD and the corresponding myocardial *DYRK1B* expression (Figure 2D, r^2^ = 0.125, *p* = 0.001).

Besides the echocardiographic data, we found several other heart failure parameters pathologically altered in patients with elevated myocardial *DYRK1B* levels. The diameter of the vena cava inferior, an indicator of volume overload, was slightly increased in patients with elevated *DYRK1B* levels (Table 1). In both cohorts, the mean vena cava inferior diameter remained within normal limits, likely because our patients were in a compensated state and received diuretic therapy prior to their elective cardiac surgery. We also found a trend toward higher NYHA classes in patients with an increased *DYRK1B* expression (*p* = 0.08 for NYHA class IV), suggesting higher heart failure symptoms. As frequently observed in patients with heart failure [21], the mean levels of C-reactive protein were significantly higher in patients with elevated *DYRK1B* expression levels (Table 1).

Notably, heart failure is mainly characterized by left ventricular dysfunction, and we analyzed the *DYRK1B* expression in atrial biopsies due to the limited availability of ventricular samples. For 6 patients, we had both right atrial and left ventricular myocardial samples for experimental analyses, which revealed comparable *DYRK1B* expression levels (Supplemental Figure S1).

### 3.3. Increased DYRK1B Expression in Patients with SDB

As SDB is highly prevalent in cardiovascular disease [22], we performed nocturnal polygraphy in all patients. This revealed that SDB was twice as common in patients with an elevated *DYRK1B* expression and was detected in 65% of these patients (*p* < 0.001 vs. patients with a low *DYRK1B* expression, Table 2). Accordingly, the mean apnea–hypopnea index (AHI) in patients with an elevated *DYRK1B* expression was almost double that observed in patients with lower *DYRK1B* levels (23.5 ± 17.1 vs. 13.0 ± 10.5) (*p* < 0.001). This was mainly driven by an increased frequency of central apneas in patients with an elevated *DYRK1B* expression, as the central apnea index was nearly 3-fold higher than in patients with a low *DYRK1B* expression (*p* < 0.001). Notably, the obstructive sleep index was not significantly different between both sub-cohorts.

While the mean and minimum oxygen saturation (SpO_2_) were comparable between both cohorts, the mean oxygen desaturation index (ODI) was twice as high in patients with *DYRK1B* levels at or above the median (20.3 ± 15.8/h vs. 10.9 ± 9.8/h, *p* < 0.001). This indicates that repetitive cycles of hypoxia and reoxygenation, which are strong inductors of oxidative stress, may be critical for *DYRK1B* regulation.

**Figure 3:**
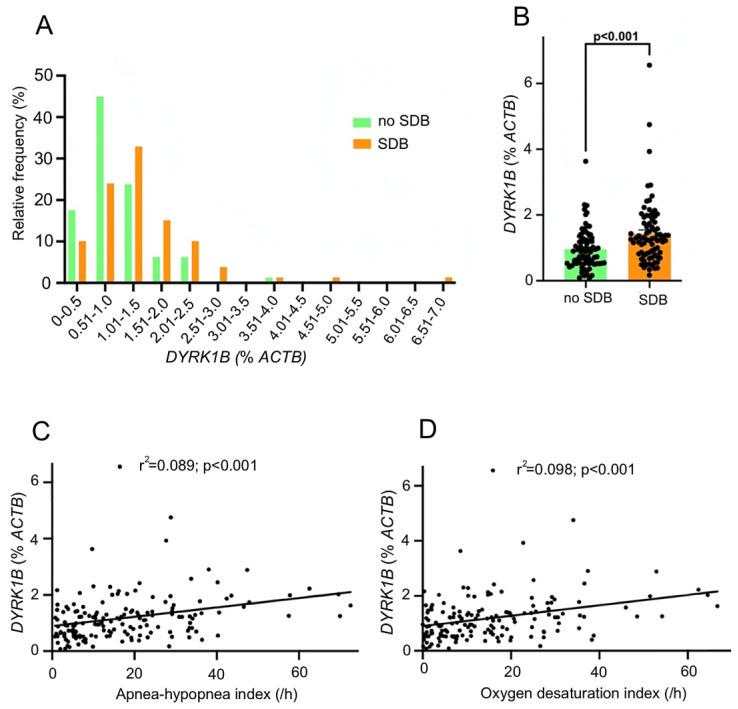
Myocardial *DYRK1B* expression is increased in patients with sleep-disordered breathing. (**A**) Frequency distribution of patients with and without sleep-disordered breathing (SDB), based on the *DYRK1B* expression. (**B**) Mean myocardial *DYRK1B* expression in patients without and with SDB. (**C**) Linear regression analysis between the apnea–hypopnea index and the corresponding *DYRK1B* expression. (**D**) Linear regression analysis between the oxygen desaturation index and the corresponding *DYRK1B* expression.

We then analyzed the frequency distribution of patients with and without SDB, based on the *DYRK1B* expression, and found a right shift toward higher *DYRK1B* expression levels in SDB patients (Figure 3A). Indeed, patients with SDB showed a 1.5-fold-increased myocardial *DYRK1B* expression (Figure 3B, *p* < 0.001). This resulted in significant positive correlations between both the ODI and the AHI with the myocardial *DYRK1B* expression (Figure 3C,D, *p* < 0.001 for both correlations).

### 3.4. Risk Analysis for an Elevated Myocardial DYRK1B Expression

**Figure 4:**
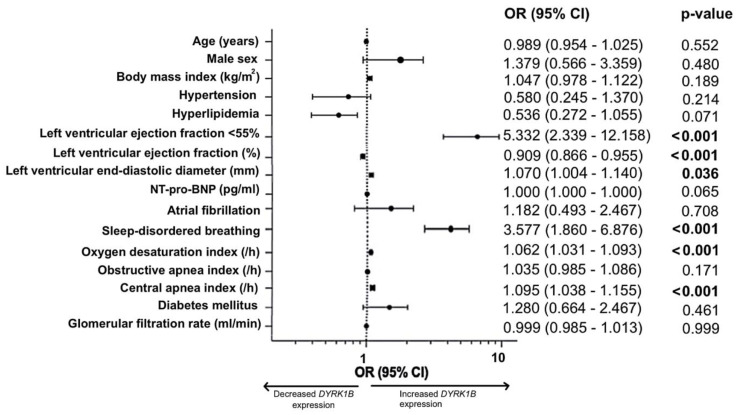
Risk analysis for an elevated myocardial *DYRK1B* expression. Forest plot showing the odds ratio (OR) and 95% confidence interval (CI) for the risk of a decrease or increase in *DYRK1B* expression, based on univariate logistic regression analyses. Statistically significant *p*-values are in bold. Abbreviation: NT-pro-BNP, N-terminal pro brain natriuretic peptide.

In an effort of extended data analysis and to investigate the relationship between *DYRK1B* expression as a binary dependent variable and defined clinical variables, we performed univariate logistic regression analyses (Figure 4). Notably, we observed a >5-fold-increased risk for an elevated *DYRK1B *expression in patients with an LVEF below 55%. The presence of SDB was linked to a 3.6-fold-increased risk for an elevated *DYRK1B* expression. Additionally, the LVEDD, an increasing ODI, and an increasing central apnea index were associated with an increased risk of *DYRK1B* upregulation.

As evident from Figure 4, there are several clinical covariates affecting the myocardial *DYRK1B* expression, which sometimes also affect each other. For instance, we observed that SDB was more prevalent in patients with a reduced LVEF, and conversely, a reduced LVEF was more common in patients with SDB. Furthermore, both the apnea–hypopnea and the oxygen desaturation index correlated significantly with the LVEF (Supplemental Figure S2).

To account for the potential confounding of these parameters, we performed a comprehensive multivariate logistic regression analysis, incorporating age, sex, LVEF, atrial fibrillation, SDB, diabetes mellitus, and the glomerular filtration rate (Table 3). Importantly, only the LVEF and SDB remained statistically significant variables in the multivariate model. Thus, even though there is a link between the LVEF and SDB, we can conclude that both variables also have an independent impact on the myocardial *DYRK1B* expression.

### 3.5. Increased Oxidative Stress Parameters in Patients with High DYRK1B Expression Levels

**Figure 5:**
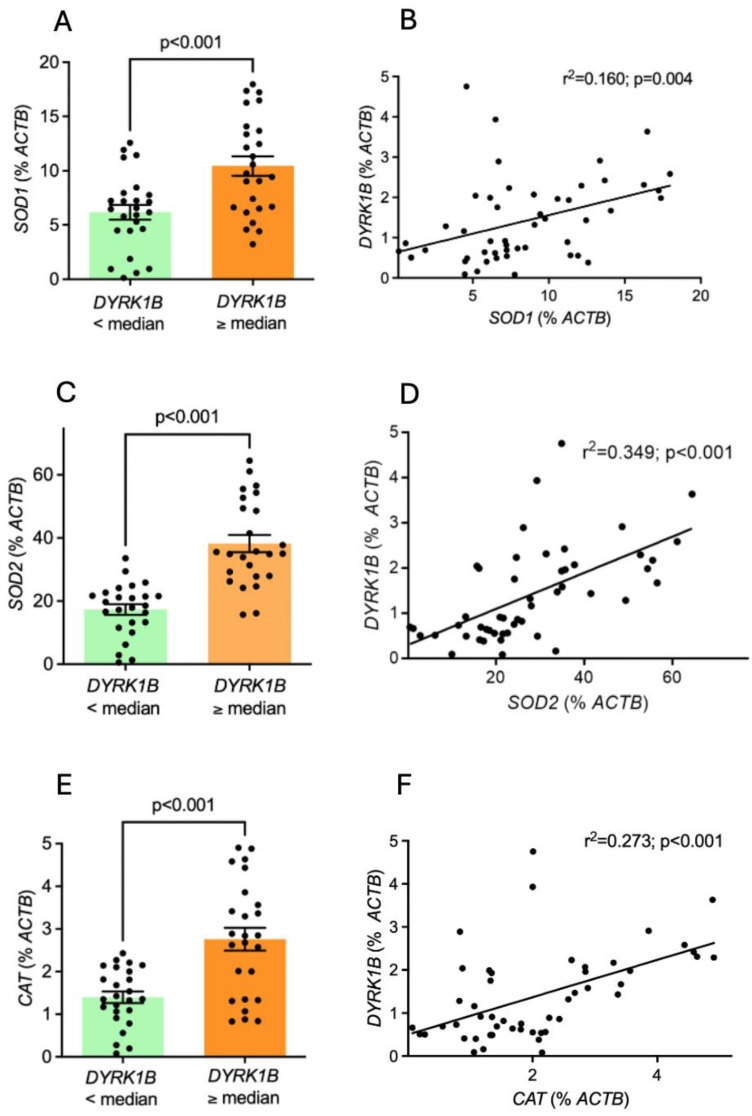
Oxidative stress parameters are elevated in patients with increased *DYRK1B* expression. (**A**) Mean *superoxide dismutase 1* (*SOD1*) expression in patients with low and high *DYRK1B* expression. (**B**) Linear regression analysis between the *SOD1* and the corresponding *DYRK1B* expression. (**C**) Mean *superoxide dismutase 2* (*SOD2*) expression in patients with low and high *DYRK1B* expression. (**D**) Linear regression analysis between the *SOD2* and the corresponding *DYRK1B* expression. (**E**) Mean *catalase* (*CAT*) expression in patients with low and high *DYRK1B* expression. (**F**) Linear regression analysis between the *CAT* and the corresponding *DYRK1B* expression.

To gain more mechanistic insights, we measured the myocardial expression levels of the oxidative stress parameters *superoxide dismutase 1* (*SOD1*), *superoxide dismutase 2* (*SOD2*), and *catalase* (*CAT*). Importantly, patients with high *DYRK1B* levels exhibited 1.5-fold higher *SOD1* levels, more than 2-fold higher *SOD2* levels, and almost twice the *CAT* levels (Figure 5A,C,E). Furthermore, all of these oxidative stress parameters were significantly positively correlated with the corresponding myocardial *DYRK1B* expression (Figure 5B,D,F).

## 4. Discussion

### 4.1. Pathomechanisms Targeted by Current Heart Failure Medications

Current heart failure medications target different cellular structures and systemic pathways aiming to improve patients’ outcome and reduce symptoms [23]. Angiotensin converting enzyme (ACE) inhibitors, angiotensin II receptor blockers (ARBs), and mineralocorticoid receptor antagonists are designed to suppress the renin–angiotensin–aldosterone system [24]. By blocking the enzyme that converts angiotensin I into the vasoconstrictive angiotensin II, ACE inhibitors reduce blood pressure and cardiac workload, whereas ARBs prevent angiotensin II from binding to its receptors, particularly in patients intolerant to ACE inhibitors [25]. By inhibiting the aldosterone receptor and reducing Na^+^ and water retention while preserving potassium, mineralocorticoid receptor antagonists prevent remodeling and fibrosis in the heart [26]. Another class of heart failure medications include beta-blockers, which act by inhibiting β-adrenergic receptors, primarily β1-receptors in the heart, which leads to a reduction in the cardiac oxygen demand and prevents cardiac remodeling [27].

More recently, inhibitors of the Na^+^–glucose transport protein 2 (SGLT2) were added to the treatment regimen of heart failure patients, as it was shown to improve cardiovascular morbidity and mortality [28]. The EMPA-REG OUTCOME trial, led by Zinman et al., showed that the SGLT2 inhibitor empagliflozin reduces the risk of cardiovascular death as well as the risk of all-cause mortality and hospitalization for heart failure [29]. However, the exact mode of action of SGLT2 inhibitors is still elusive. Initially, the gliflozins where thought to be specific blockers of SGLT2 in the proximal tubule of the kidney, leading to a reduced renal glucose reabsorption and enhanced urinary glucose excretion [29]. Recent studies indicate that SGLT2 inhibitors also modulate inflammation and fibrosis through mechanisms that include reduction in uric acid and modulation of cytokine production [30]. Further reported mechanisms are a reduced plasma volume due to natriuretic effects as well as decreased oxidative stress and improved mitochondrial function [31,32]. Emerging research suggests that SGLT2 inhibitors may also have direct cardiac effects. An excellent study led by Mustroph et al. recently demonstrated that empagliflozin blocks the cardiac stress-responsive enzyme Ca^2+^/calmodulin-dependent protein kinase IIδ (CaMKIIδ) resulting in reduced sarcoplasmic reticulum (SR) Ca^2+^ leakage and protection against heart failure-related cellular dysfunction [33,34].

The above-discussed and currently used heart failure medications target important cellular structures and signaling cascades to silence cardiovascular pathomechanisms. Noteworthy, certain signaling cascades remain unexplored and untargeted, like the DYRK1B pathway. As a part of the dual-specificity tyrosine-phosphorylation-regulated kinase family, this protein plays a central role in modulating cell cycle progression and metabolic adaption [35,36,37].

### 4.2. Pathomechanisms in Sleep-Disordered Breathing

Sleep-disordered breathing (SDB) represents an underestimated global health issue, with the number of people affected exceeding one billion worldwide [38]. Typical symptoms of SDB include daytime sleepiness and impairments in cognition and performance [39]. Untreated SDB is associated with a high risk of cardiovascular disease [40]. SDB was linked to arterial hypertension, atrial arrhythmias that favor thrombogenesis, and subsequent strokes [41,42,43,44]. Plus, approximately 50% of heart failure patients also have SDB [45,46]. Notably, patients with heart failure with reduced ejection fraction (HFrEF) have more frequently central apneas [45]. Our findings are in line with these clinical observations. While *DYRK1B* expression correlated negatively with the left ventricular ejection fraction, we also found a significant correlation with the central apnea index but not with the obstructive apnea index (Table 2).

SDB can be effectively treated with continuous airway pressure (CPAP), mandibular advancement splints, hypoglossus nerve stimulation, and life-style interventions, particularly by weight reduction [47]. However, patients’ compliance to lifestyle interventions like diet or exercise is often low [48,49,50]. Thus, current therapeutic strategies are typically based on continuous airway pressure (CPAP) therapy [47]. Notably, CPAP therapy recently failed to improve the cardiovascular outcome or the burden of atrial fibrillation [51,52]. This could be due to a limited treatment effectiveness but also due to a poor patients’ compliance to CPAP therapy that is typically observed in patients with cardiovascular disease [53]. Recently, it has been demonstrated that patients with obstructive sleep apnea and obesity treated with tirzepatide led, among other outcomes, to weight loss, a reduction in AHI and hsCRP, positioning tirzepatide as a promising potential treatment approach for this patient group in the future [54]. For patients with central SDB, it is even more difficult to give treatment recommendations, as it was recently found in the SERVE-HF trial that adaptive servo-ventilation increases all-cause and cardiovascular mortality in HFrEF patients, who typically show central apneas [55].

SDB is a systemic disorder with key features like intermittent hypoxia, intrathoracic pressure swings, and increased β-adrenergic stress that all dysregulates a multitude of processes [42,56,57,58]. This includes oxidative stress, metabolic alterations, and inflammation leading to structural remodeling and fibrosis [20,42,56,57]. Another central driver for myocardial dysregulation in SDB is a pathological overactivation of the stress-responsive enzyme CaMKIIδ [46,59,60]. We found increased levels of reactive oxygen species (ROS) in the myocardium of patients and mice with SDB that facilitated CaMKIIδ oxidation at two critical methionines [46,59,60,61,62]. Oxidation of CaMKIIδ releases the catalytic domain, leading to enzyme hyperactivation and a pathological dysregulation of the myocardial Na^+^ and Ca^2+^ homeostasis [58,62]. Specifically, we found an enhanced late Na^+^ current with an increased sarcoplasmic reticulum Ca^2+^ leakage, leading to impaired Ca^2+^ transients, which all culminated in cardiac contractile dysfunction and arrhythmias [46,59,60,61,62].

In our present study, we found *DYRK1B* expression highly upregulated in the myocardium of SDB patients. One may argue that this could be a coincidence, as the correlation with SDB was mainly driven by central apneas, which are typically found in patients with a low LVEF, which also correlated with the *DYRK1B* expression. In addition, we also observed an interdependence between the LVEF and SDB in our patient population. To account for this, we performed a multivariate logistic regression analysis incorporating—among other important clinical covariates—both the LVEF and SDB. Notably, both parameters remained statistically significant in the multivariate model, indicating that they both have an impact on myocardial *DYRK1B* expression [63].

### 4.3. DYRK1B as a Potential Therapeutic Target for Cardiovascular Disease

Up to now, the impact of DYRK1B on heart function is a little-researched area. Zhuang et al. demonstrated an upregulation of DYRK1B expression not only in hypertrophic mouse hearts but also in myocardial biopsies of five patients with dilated cardiomyopathy [16]. Plus, the authors reanalyzed previously published RNA-sequencing datasets and found that *DYRK1B* upregulated in the ventricular myocardium of hypertrophic, dilative, and ischemic cardiomyopathy [16]. Unfortunately, there were no other clinical data available, which complicates clinical interpretation. In the present study, we quantified *DYRK1B* expression in myocardial atrial biopsies of 159 very well-characterized cardiovascular patients, including a multivariate regression analysis with important clinical parameters and potential confounders. Plus, we quantified the *DYRK1B* expression with real-time qPCR, which is targeted and more accurate than reanalyses of preexisting RNA-sequencing datasets. Therefore, we were able to verify and expand upon the findings of Zhuang et al. in a larger and better-characterized patient cohort.

Cardiac-specific overexpression of DYRK1B was reported to impair LVEF and increase cardiac fibrosis in mice [16]. In contrast, *DYRK1B* knockout mice were cardioprotected from afterload-induced cardiac hypertrophy and contractile dysfunction [16]. This was based on impaired mitochondrial bioenergetics, as DYRK1B was found to bind directly to STAT3, thereby increasing its phosphorylation [16]. This led to a downregulation of the peroxisome proliferator-activated receptor gamma coactivator-1α (PGC-1α), a master regulator of energy metabolism [16]. In our study, we found that myocardial *DYRK1B* expression did not correlate with mean or minimum oxygen saturation but with the oxygen desaturation index. This indicates that cyclic fluctuations in oxygen levels, which is a strong inductor of oxidative stress [12], could be a key driver of *DYRK1B* regulation [64]. To test this, we quantified the myocardial expression of *superoxide dismutase 1* (*SOD1*), *superoxide dismutase 2* (*SOD2*), and *catalase* (*CAT*), which are well-established markers of oxidative stress [65,66,67]. Indeed, all these parameters of oxidative stress were significantly increased in patients with a high *DYRK1B* expression, and we also detected positive correlations between these markers and the corresponding *DYRK1B* expression.

### 4.4. Challenges of Targeting DYRK1B

As discussed above, DYRK1B represents a promising molecular target to tackle heart failure from another angle [16]. However, compound-based enzyme inhibition faces several challenges and limitations [68]. First, there are several DYRK isoforms, some even with opposing functions [9]. While DYRK1B is linked to an impaired myocardial function, DYRK1A is critically involved in neuronal processes, including neuronal migration, dendrite formation, and synaptic functionality [69]. Additionally, emerging research highlights that DYRK2 plays significant roles in several cellular processes, particularly by regulating cellular homeostasis and modulating autophagy through phosphorylation of critical substrates, such as p53 [11]. This means that an unspecific DYRK blockade would cause unforeseeable side effects, potentially ranging from disturbances in neuronal function to impairments in regulating cellular processes and immune responses. Unfortunately, the various DYRKs are structurally highly similar, especially in the catalytic domain where an ATP-competitive compound would bind. For instance, DYRK1A and DYRK1B share 85% homology, classifying them as closely related paralogous kinases [12]. This underlines the difficulty of developing an inhibitor with high specificity for DYRK1B.

However, a traditional pharmacological compound, even with a high DYRK1B specificity, would block the enzyme also in other tissues, where DYRK1B is not necessarily pathogenic. There are several other tissues with a significant protein expression, like in neurons, the lungs, the kidneys, and skeletal muscles [70]. DYRK1B plays a pivotal role in stem cell biology, being crucial for regulating the balance between proliferation and differentiation of stem cells during processes such as spermatogenesis and neurogenesis [37]. It contributes to skeletal muscle differentiation and function by mediating key signaling pathways [71]. Therefore, blocking DYRK1B systemically is very likely to entail significant adverse side effects like neuronal or skeletal muscle dysfunction. Interestingly, it was recently reported in patients that hereditary DYRK1B mutations with a complete loss-of-function of DYRK1B (in all tissues) was associated with a substantially increased risk of obesity (odds ratio of 8) and type 2 diabetes [72]. This indicates that it is critical to target specifically DYRK1B and exclusively in one tissue to keep the risk of adverse side effects at a minimum.

However, even a perfect compound with high isoform and tissue specificity would mean an additional pill for the daily treatment regimen of a heart failure patient. Unfortunately, every extra pill decreases the patients’ compliance to take their prescribed medication. This is a central issue in cardiovascular medicine, as it decreases treatment success and represents a dramatic economic burden [73,74,75]. Therefore, new and advanced biotechnological tools are required to overcome these challenges of DYRK1B inhibition [76].

## 5. Limitations

The analyzed patient cohort was highly selective, as all participants were patients diagnosed with coronary artery disease undergoing elective coronary artery bypass grafting (CABG). This limits the generalizability of our findings to different patient populations.

Even though we analyzed myocardial samples of 159 patients, the sample size was relatively small in some subgroups. For instance, only four patients were in the subgroup with an LVEF < 30. Because small sample sizes are more susceptible to variability bias, this could explain why we did not detect a further *DYRK1B* increase in the subgroup with the lowest LVEF.

We assessed the gene expression levels of important oxidative stress parameters using qPCR. Even though this is a valid approach to investigate oxidative stress, it is not a direct measure of reactive oxygen species (ROS). In future studies, we will aim to obtain fresh myocardial biopsies for live cell imaging and ROS measurements using confocal laser scanning microscopy [77].

## 6. Conclusions

In our study, we quantified the myocardial *DYRK1B* expression in 159 cardiovascular high-risk and clinically very well-characterized patients undergoing elective coronary artery bypass grafting. We found that *DYRK1B* was highly upregulated in patients with impaired cardiac contractility or sleep-disordered breathing. Even though there was an interdependence between the LVEF and SDB, multivariate regression analysis revealed that both entities also had their own impact on myocardial *DYRK1B* expression. Thus, DYRK1B appears to be a promising molecular target for developing new therapeutic strategies against heart failure and potentially SDB, which will be tested in future studies. Considering the DYRK1B-signaling pathway as a new potential point of intervention, further research is needed to gain better understanding of the underlying mechanisms and whether it conveys additional therapeutic benefits compared to current heart failure medications. Given the structural similarity of DYRK1B to other proteins (especially of the DYRK family) and its ubiquitous expression in the human body, target- and tissue-specificity will be important, which requires new and advanced biotechnological tools.

## Figures and Tables

**Table 1:** Baseline characteristics of patients undergoing elective coronary artery bypass grafting.

	Total Cohort (n = 159)	*DYRK1B* < Median (n = 79)	*DYRK1B* ≥ Median (n = 80)	*p*-Value
**Age (years)**	66.1 ± 8.7	66.5 ± 9.2	65.7 ± 8.2	0.555 ^T^
**Male sex**	136 (86)	66 (84)	70 (88)	0.507 ^Chi^
**Body Mass Index (kg/m^2^)**	28.5 ± 4.6	28.0 ± 4.4	29.0 ± 4.8	0.188 ^T^
**Diabetes mellitus**	62 (39)	29 (37)	33 (41)	0.460 ^Chi^
**Hyperlipidemia**	108 (68)	59 (75)	49 (61)	0.070 ^Chi^
**Hypertension**	133 (84)	69 (87)	64 (80)	0.211 ^Chi^
**Atrial fibrillation**	24 (15)	11 (14)	13(16)	0.708 ^Chi^
**Heart function**				
** NYHA functional class**				
** I**	44 (28)	21 (27)	23 (29)	0.761 ^Chi^
** II**	65 (41)	35 (44)	30 (38)	0.384 ^Chi^
** III**	47 (30)	23 (29)	24 (30)	0.903 ^Chi^
** IV**	3 (2)	0 (0)	3 (4)	0.083 ^Chi^
** Left ventricular ejection raction (LVEF) (%)**	54.1 ± 10.4	58.4 ± 5.9	50.6 ± 11.9	**<0.001 ^W^**
** LVEF, vis. assessment**	1.6 ± 0.8	1.2 ± 0.4	1.9 ± 1.0	**<0.001 ^W^**
** Left ventricular end-diastolic diameter (mm)**	52.1 ± 6.9	50.6 ± 6.6	53.6 ± 6.9	**0.019 ^W^**
** Left ventricular end-systolic diameter (mm)**	38.0 ± 9.7	36.4 ± 10.2	40.0 ± 8.6	0.106 ^T^
** Canadian Cardiovascular Society (CCS)**				
** Class 0**	27 (17)	8 (10)	19 (24)	**0.023 ^Chi^**
** Class 1**	26 (16)	13 (16)	13 (16)	0.972 ^Chi^
** Class 2**	58 (36)	31 (39)	27 (35)	0.472 ^Chi^
** Class 3**	46 (29)	26 (33)	20 (25)	0.271 ^Chi^
** Class 4**	3 (2)	1 (1)	2 (3)	0.567 ^Chi^
**Coronary artery status**				
** Number of stenoses**	3.9 ± 1.8	3.6 ± 1.4	4.1 ± 2.0	0.073 ^T^
** Number of arterial grafts**	1.2 ± 0.6	1.2 ± 0.5	1.1 ± 0.6	0.222 ^W^
** Number of venous grafts**	1.4 ± 0.75	1.3 ± 0.8	1.4 ± 0.7	0.406 ^W^
**Systolic pulmonary artery pressure (mmHg)**	26.2 ± 11.2	23.6 ± 11.2	28.2 ± 11.0	0.103 ^T^
**Vena cava inferior (mm)**	15.3 ± 4.05	14.3 ± 3.4	16.5 ± 4.5	**0.011 ^T^**
**NT-pro-BNP (pg/mL)**	1285.2 ± 3281.0	781.4 ± 1325.2	1835.5 ± 4494.2	0.111 ^W^
**Glomerular filtration rate (mL/min)**	73.2 ± 22.4	73.5 ± 22.8	73.0 ± 22.3	0.863 ^W^
**Cholesterol (mg/dL)**	193.4 ± 48.2	200.2 ± 48.4	175.7 ± 44.9	0.136 ^T^
**Low-density lipoprotein (mg/dL)**	126.0 ± 49.7	130.1 ± 49.4	115.9 ± 51.3	0.412 ^T^
**C-reactive protein (mg/L)**	7.8 ± 16.4	6.1 ± 10.8	9.4 ± 20.4	**0.039 ^W^**
**Patient treatment**				
** Angiotensin-converting enzyme inhibitors**	80 (50)	43 (54)	37 (46)	0.652 ^Chi^
** Angiotensin receptor blockers**	39 (25)	21 (27)	18 (23)	0.794 ^Chi^
** Calcium channel blockers**	40 (25)	23 (29)	17 (21)	0.418 ^Chi^
** Beta-blockers**	98 (62)	48 (61)	50 (63)	0.288 ^Chi^
** Mineralocorticoid receptor antagonists**	14 (9)	5 (6)	9 (11)	0.198 ^Chi^
** Loop diuretics**	41 (26)	19 (24)	22 (28)	0.388 ^Chi^
** Thiazide diuretics**	27 (17)	12 (15)	15 (19)	0.381 ^Chi^
** Statins**	116 (73)	66 (84)	50 (63)	**0.021 ^Chi^**

Data are presented as mean ± standard deviation or as total number of patients (with relative proportion). Statistically significant *p*-values are in bold. Abbreviations: Chi, Chi-square test; *DYRK1B*, dual-specificity tyrosine-regulated kinase 1B; LVEF vis. assessment, visual assessment divided in 1: EF ≥ 55%, 2: ≥45–54%, 3: ≥30–44% and 4: <30%; NT-pro-BNP, N-terminal pro brain natriuretic peptide; NYHA, New York Heart Association; T, Student’s *t* test; W, Wilcoxon-Mann–Whitney test.

**Table 2:** Nocturnal polygraphy data.

	Total Cohort (n = 159)	*DYRK1B* < Median (n = 79)	*DYRK1B* ≥ Median (n = 80)	*p*-Value
**Total recording time (min)**	481.9 ± 47.6	480.8 ± 54.6	483.0 ± 38.5	0.516 ^W^
**Sleep-disordered breathing**	80 (50)	28 (35)	52 (65)	**<0.001 ^Chi^**
**Apnea–hypopnea index (/h)**	18.3 ± 15.1	13.0 ± 10.5	23.5 ± 17.1	**<0.001** ^W^
**Obstructive apnea index (/h)**	4.9 ± 7.2	4.1 ± 5.7	5.7 ± 8.4	0.122 ^W^
**Central apnea index (/h)**	5.7 ± 8.4	3.3 ± 5.0	8.2 ± 10.2	**<0.001** ^W^
**Oxygen desaturation index (/h)**	15.6 ± 14.0	10.9 ± 9.8	20.3 ± 15.8	**<0.001** ^W^
**Mean oxygen saturation (%)**	92.3 ± 2.4	92.5 ± 2.5	92.1 ± 2.4	0.141 ^W^
**Minimum oxygen saturation (%)**	81.0 ± 6,8	81.7 ± 7.1	80.3 ± 6.5	0.104 ^W^
**Time of SpO_2_ < 90%/TRT (%)**	14.3 ± 20.0	11.5 ± 18.6	16.9 ± 21.1	**0.049 ^W^**

Data are presented as mean ± standard deviation or as total number of patients (with relative proportion). Statistically significant *p*-values are in bold. SDB is defined as AHI ≥ 15/h. Abbreviations: SpO_2_, oxygen saturation of arterial blood; TRT, total recording time; W, Wilcoxon–Mann–Whitney test; Chi, Chi-square test.

**Table 3:** The left ventricular ejection fraction and sleep-disordered breathing are independent predictors of myocardial *DYRK1B* expression in the multivariate regression analysis.

	OR (95% CI)	*p*-Value
Multivariate model for an increased *DYRK1B* expression r^2^ = 0.265; *p* < **0.001**		
Age (years)	1.005 (0.957–1.055)	0.847
Male sex	0.744 (0.228–2.433)	0.625
Body mass index (kg/m^2^)	1.022 (0.933–1.119)	0.645
Left ventricular ejection fraction (%)	0.914 (0.865–0.965)	**0.001**
Atrial fibrillation	0.855 (0.283–2.590)	0.782
Sleep-disordered breathing	2.923 (1.253–6.822)	**0.013**
Diabetes mellitus	0.495 (0.192–1.272)	0.144
Glomerular filtration rate (mL/min/1.73 m^2^)	1.007 (0.987–1.027)	0.509

Multivariate logistic regression analysis incorporating important clinical covariates that potentially affect *DYRK1B* expression. Statistically significant *p*-values are in bold. Abbreviations: CI, confidence interval; OR, odds ratio.

## Data Availability

Data are contained within the article.

## Supplementary Materials

The following supporting information can be downloaded at https://www.mdpi.com/article/10.3390/antiox14020163/s1: Figure S1: Mean DYRK1B expression is comparable in the right atrium and left ventricle within the same patients; Figure S2: Interdependence between LVEF and SDB; Table S1: Additional baseline characteristics of patients undergoing elective coronary artery bypass grafting, based on the LVEF and the presence of SDB; Table S2: Postoperative patient outcome.
