# Supplementary material for: Myocardial DYRK1B Expression Is Increased in Patients with Impaired Cardiac Contractility and Sleep-Disordered Breathing
Source: Antioxidants (Basel). 2025 Jan 29;14(2):163. doi: 10.3390/antiox14020163 (PMC11851367; doi:10.3390/antiox14020163)
Supplement: Supplementary file 1 [file antioxidants-14-00163-s001.zip › antioxidants-3399703-supplementary.pdf]

Supplementary Materials

Myocardial *DYRK1B* expression is increased in patients with impaired cardiac contractility and sleep-disordered breathing

Fatma Bayram[1], Philipp Hegner[1], Anna-Maria Lauerer[1], Sönke Schildt[1], Dominik Wermers[1], Maria Johanna Baier[1], Julian Mustroph[1], Maria Tafelmeier[1], Zdenek Provaznik[2], Christof Schmid[2], Lars Siegfried Maier[1], Stefan Wagner[1], Michael Arzt[1], and Simon Lebek[1,*]

[1] Department of Internal Medicine II, University Hospital Regensburg, Regensburg, Germany
[2] Department of Cardiothoracic Surgery, University Hospital Regensburg, Regensburg, Germany
* Correspondence: simon.lebek@ukr.de

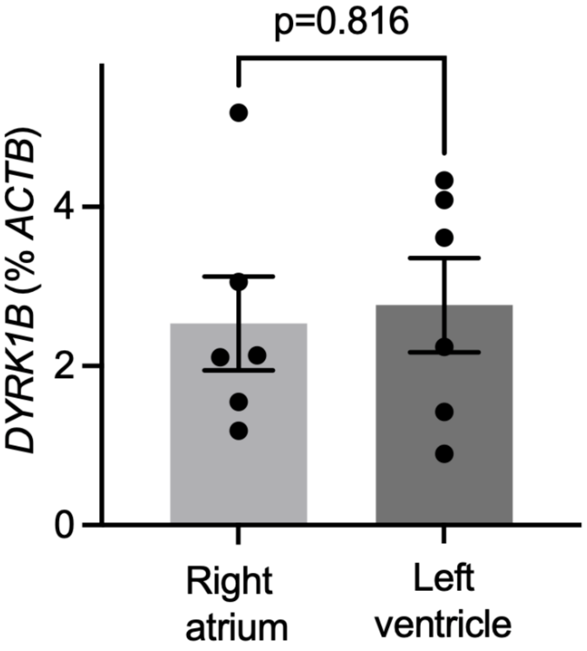

Figure S1 Mean *DYRK1B* expression is comparable in the right atrium and left ventricle within the same patients.

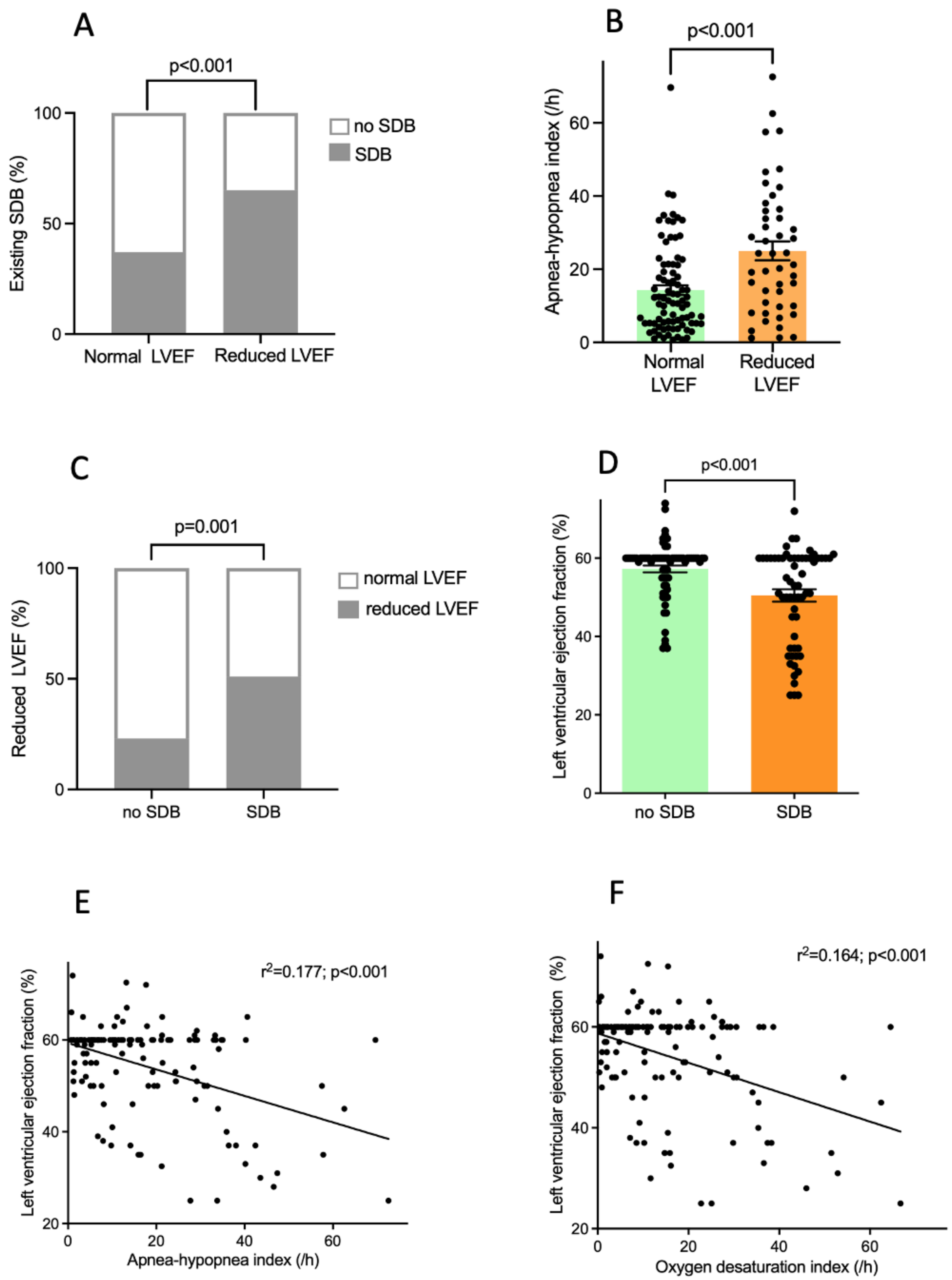

Figure S2 Interdependence between LVEF and SDB.
(A) Mean proportion of patients with a normal and a reduced (<55%) left ventricular ejection fraction (LVEF) having sleep-disordered breathing (SDB). **(B)** Mean apnea-hypopnea index in patients with a normal and a reduced LVEF. **(C)** Mean proportion of patients without and with SDB having a reduced LVEF. **(D)** Mean LVEF in patients without and with SDB. **(E)** Linear regression analysis between the apnea-hypopnea index and the LVEF. **(F)** Linear regression analysis between the oxygen desaturation index and the LVEF.

	LVEF ≥ 55% (n=83)		LVEF < 55% (n=47)		p-value
	**No SDB (n=53)**	**SDB (n=30)**	**No SDB (n=16)**	**SDB (n=31)**	
***DYRK1B* expression (% *ACTB*)**	0.9 ± 0.4	1.0 ± 0.5	1.4 ± 1.0	2.1 ± 1.2	**<0.001[We]**
**Age (years)**	65.0 ± 9.0	67.5 ± 6.9	64.1± 10.1	65.5 ±8.8	0.559[A]
**Male sex**	44 (83)	26 (87)	14 (88)	28 (90)	0.821[Chi]
**Body Mass Index (kg/m^2)**	27.1 ± 4.2	29.5 ± 5.2	28.4 ± 4.3	29.8 ± 4.6	**0.033[A]**
**Diabetes mellitus**	18 (34)	12 (40)	7 (15)	13 (42)	**0.032[Chi]**
**Hyperlipidemia**	35 (66)	21 (70)	12 (26)	21 (68)	0.917[Chi]
**Left ventricular ejection fraction (LVEF) (%)**	60.5 ± 3.5	60.6 ± 3.0	46.4 ± 6.0	41.0 ± 1.7	**<0.001[A]**
**Left ventricular end-diastolic diameter (mm)**	48.6 ± 6.0	51.3 ± 5.3	52.4 ± 8.1	57.0 ± 5.9	**0.001[We]**
**NT-pro BNP (pg/ml)**	411.5 ± 511.8	772.7 ± 1079.2	1657.7 ± 2612.9	3187.7 ± 6620.9	**0.040[We]**
**Vena cava inferior (mm)**	15.3 ± 4.0	14.0 ± 3.8	16.8 ± 4.4	18.3 ± 4.3	0.059[A]
**Glomerular filtration rate (ml/min)**	76.0 ± 23.0	71.9 ± 20.2	73.8 ± 20.0	67.4 ± 26.0	0.435[A]
**C-reactive protein (mg/l)**	6.0 ± 11.8	4.1 ± 3.3	8.1 ± 18.2	14.8 ± 28.8	0.147[We]
**Total recording time (min)**	473.9 ± 51.8	490.4 ± 28.6	502.3 ± 23.4	468.2 ± 47.1	**0.003[A]**
**Apnea-hypopnea index (/h)**	7.0 ± 4.2	27.5 ± 10.9	7.0 ± 4.2	33.7 ± 14.6	**<0.001[We]**
**Obstructive apnea index (/h)**	1.6 ± 1.9	9.6 ± 7.5	1.1 ± 1.3	6.9 ± 10.1	**<0.001[We]**
**Oxygen desaturation index (/h)**	5.8 ± 4.3	23.5 ± 11.1	6.9 ± 4.7	30.0 ± 15.6	**<0.001[We]**
**Mean oxygen saturation (%)**	92.8 ± 1.8	92.7 ± 1.5	93.0 ± 3.9	91.4 ± 2.4	**0.023[We]**
**Time of SpO_2 <90%/TRT (%)**	8.3 ± 14.5	10.1 ± 10.5	16.7 ± 28.4	23.7 ± 22.3	**0.002[We]**
**Patient treatment**					
**Angiotensin-converting enzyme inhibitors**	29 (55)	16 (53)	11 (69)	14 (45)	0.551[Chi]
**Angiotensin receptor blockers**	13 (16)	6 (20)	1 (1)	10 (32)	0.155[Chi]
**Calcium channel blockers**	16 (30)	7 (23)	5 (31)	9 (29)	0.816[Chi]
**Beta-blockers**	33 (62)	21 (70)	7 (44)	22 (71)	0.136[Chi]
**Mineralocorticoid receptor antagonists**	2 (4)	2 (7)	3 (19)	4 (13)	0.168[Chi]
**Loop diuretics**	8 (15)	5 (17)	4 (25)	12 (39)	**0.030[Chi]**
**Thiazide diuretics**	8 (15)	8 (27)	3 (19)	6 (19)	0.712[Chi]
**Statins**	41 (77)	24 (80)	13 (81)	20 (65)	0.767[Chi]

Table S1 Additional baseline characteristics of patients undergoing elective coronary artery bypass grafting, based on the LVEF and the presence of SDB.

Data are presented as mean ± standard deviation or as total number of patients (with relative proportion). Abbreviations: A, one-way ANOVA; Chi, Chi-square test; *DYRK1B, dual-specificity tyrosine-regulated kinase 1B*; NT-pro-BNP, N-terminal pro brain natriuretic peptide; TRT, total recording time; We, Welch's test; W, Wilcoxon-Mann-Whitney test.

	Total cohort (n=159)	*DYRK1B* < median (n=79)	*DYRK1B* ≥ median (n=80)	p-value
**Prolonged mechanical ventilation (>24h)**	14 (9)	10 (13)	4 (5)	0.089[Chi]
**Postoperative hypoxemia (SaO_2 <90% or desaturation >4% perioperative)**	46 (29)	20 (25)	26 (33)	0.342[Chi]
**Postoperative atrial fibrillation**	24 (15)	9 (9)	15 (19)	0.195[Chi]
**Occurrence of major adverse cardiac cerebrovascular events 0-7 days postoperative**	12 (8)	4 (5)	8 (10)	0.191[F]
**Length of postoperative stay on ICU (d)**	2.6 ± 2.5	2.5 ± 2.3	2.6 ± 2.8	0.793[T]
**Length of postoperative stay in hospital (d)**	12.4 ± 7.7	11.4 ± 6.0	13.3 ± 8.9	0.120[T]

Table S2 Postoperative patient outcome.

Data are presented as total number of patients (with relative proportion) or as mean ± standard deviation. Abbreviation: Chi, Chi-square test; F, Fisher's exact test; T, Student's t test.
